# The Role of the Effects of Autophagy on NLRP3 Inflammasome in Inflammatory Nervous System Diseases

**DOI:** 10.3389/fcell.2021.657478

**Published:** 2021-05-17

**Authors:** Shizhen Zhao, Xiaotian Li, jie Wang, Honggang Wang

**Affiliations:** Henan International Joint Laboratory of Nuclear Protein Regulation, School of Basic Medical Sciences, Henan University, Kaifeng, China

**Keywords:** autophagy, NLRP3 inflammasome, Alzheimer’s disease, Parkinson’s disease, depression

## Abstract

Autophagy is a stable self-sustaining process in eukaryotic cells. In this process, pathogens, abnormal proteins, and organelles are encapsulated by a bilayer membrane to form autophagosomes, which are then transferred to lysosomes for degradation. Autophagy is involved in many physiological and pathological processes. Nucleotide-binding oligomerization domain-like receptor protein 3 (NLRP3) inflammasome, containing NLRP3, apoptosis-associated speck-like protein containing a caspase recruitment domain (ASC) and pro-caspase-1, can activate caspase-1 to induce pyroptosis and lead to the maturation and secretion of interleukin-1 β (IL-1 β) and IL-18. NLRP3 inflammasome is related to many diseases. In recent years, autophagy has been reported to play a vital role by regulating the NLRP3 inflammasome in inflammatory nervous system diseases. However, the related mechanisms are not completely clarified. In this review, we sum up recent research about the role of the effects of autophagy on NLRP3 inflammasome in Alzheimer’s disease, chronic cerebral hypoperfusion, Parkinson’s disease, depression, cerebral ischemia/reperfusion injury, early brain injury after subarachnoid hemorrhage, and experimental autoimmune encephalomyelitis and analyzed the related mechanism to provide theoretical reference for the future research of inflammatory neurological diseases.

## Introduction

**FIGURE 1:**
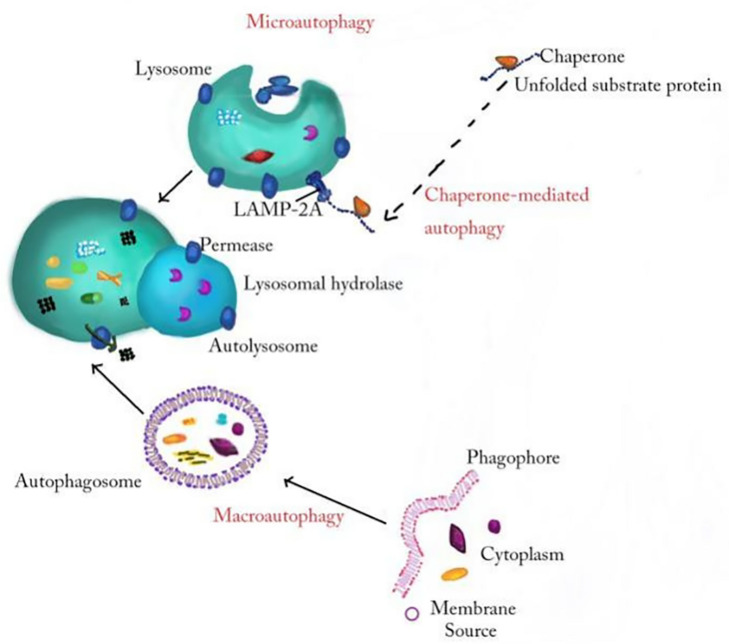
The general processes of macroautophagy, microautophagy, and chaperone-mediated autophagy. In the process of macroautophagy, the inclusion is wrapped by a double membrane structure to form an autophagosome and then fuses with a lysosome to degrade. Microautophagy refers to the direct invagination of the lysosomal membrane and the encapsulation of the cell contents. In chaperone-mediated autophagy, cytoplasmic proteins combine with chaperones, and are transported to lysosomal chambers and then digested by lysosomal enzymes.

Autophagy, which is a closely coordinated process, isolates aged/damaged organelles and misfolded and mutated proteins into bilayer membrane vesicles named autophagosomes and then fuses into lysosomes, leading to the degradation of isolated components (Lv et al., 2021). Autophagy can be divided into macroautophagy, microautophagy, and chaperone-mediated autophagy according to the inducing signal, action time, target type, and transport pathway into lysosomes. Macroautophagy involves the formation of a double membranous vesicle that isolates the cytoplasm. The complete vesicles, called autophagosomes, then fuse with lysosomes for subsequent degradation (Wang et al., 2019a; Zhu et al., 2019). In microautophagy, the substances destined for degradation reach lysosome cavity through invagination of lysosome or endoplasmic membrane (Sahu et al., 2011). Chaperone-mediated autophagy occurs only in mammalian cells, allowing selective degradation of proteins with specific amino acid sequences (Figure 1; Kaushik and Cuervo, 2012). Among these three autophagy processes, macroautophagy, referred to as autophagy, is the most active form and has been widely studied in diseases (Ueno and Komatsu, 2017; Galluzzi and Green, 2019). Beclin1, LC3, P62, and other conserved proteins participate in the autophagy process and are regarded as autophagy-related proteins (Wang et al., 2019a). Among them, LC3, a ubiquitin-like protein, promotes autophagosome formation (Fujita et al., 2008; Pyo et al., 2012). It regulates the elongation and closure of the autophagic membrane by binding with phosphatidylethanolamine (Ichimiya et al., 2020). Autophagy is affected by many factors, such as endoplasmic reticulum stress (ERS), immune or inflammatory stimulation, nutritional deficiency, Ca^2+^ concentration and accumulation of organelle damage (Tooze and Yoshimori, 2010; Mizushima et al., 2011). Autophagy is usually maintained at the basic level under physiological conditions. In the pathological state, the upregulated autophagy can eliminate the dysfunctional proteins in cells and help them survive (Glick et al., 2010). Autophagy is a double-edged sword because, if autophagy is maintained at a high level, autophagy leads to cell death (Liu and Levine, 2015; Garcia-Huerta et al., 2016). Many studies find that autophagy played an important role in neurodegenerative diseases (Menzies et al., 2015), cardiovascular diseases (Shirakabe et al., 2016), infection, and immunity (Deretic et al., 2013). In particular, the role of autophagy in inflammatory nervous system diseases is reported by many researchers; for example, MiR-124 inhibits the secretion of proinflammatory mediators by promoting autophagy in Parkinson’s disease (PD) (Yao et al., 2019), and the upregulation of autophagy of hippocampal cells improved memory impairment led by ethanol through an anti-inflammatory mechanism (Liu et al., 2019). The mechanism about autophagy in inflammatory nervous system diseases needs to be further studied.

**FIGURE 2:**
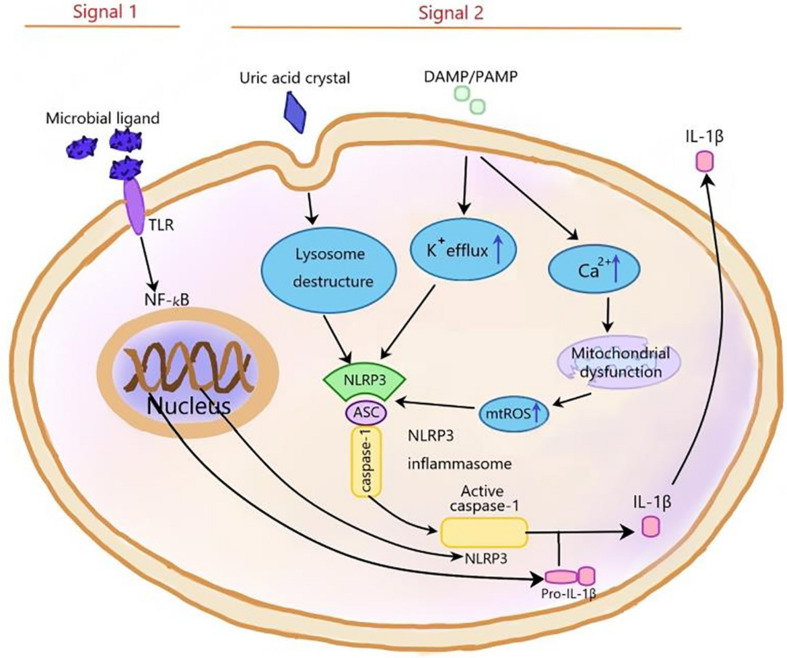
The NLRP3 inflammasome is activated by signal 1 and signal 2. Signal 1, which is mediated by microbial ligands recognized by TLR, activates NF-KB pathway to upregulate pro-IL-1β and NLRP3 expression. Signal 2, which consists of three ways, induces the assembly of NLRP3 inflammasome complex. (1) In the noninfectious condition, K^+^ efflux activates the NLRP3 inflammasome. (2) The endogenous and exogenous particles, such as uric acid crystallization, induce lysosome damage to activate NLRP3 inflammasome. (3) The increase of intracellular Ca^2+^ concentration increases mtROS level to activate NLRP3 inflammasome. NF-kB, nuclear factor kappa-light-chain-enhancer of activated B cells; ASC, apoptosis-associated speck-like protein containing a C-terminal caspase recruitment domain; ROS, reactive oxygen species; TLR, toll-like receptor.

Inflammasomes, first proposed by Martinon and coworkers in 2002 (Martinon et al., 2002), are a kind of protein cytoplasmic complex, which can activate the effective inflammatory mediators. As a part of the innate immune response against invading pathogens, inflammasomes are activated by cell infection or pressure stimulation and induce the expression, maturation, and release of a variety of proinflammatory cytokines; therefore, triggering a series of inflammatory reactions (Schroder and Tschopp, 2010; Yaribeygi et al., 2019). The nucleotide-binding oligomerization domain-like receptor (NLR) family can be divided into three subfamilies: the NLRPs (NLRP1-14), the NODs (NOD1-2, NOD3/NLRC3, NOD4/NLRC5, NOD5/NLRX1, and CIITA), and the IPAF subfamily, including NAIP and IPAF (Schroder and Tschopp, 2010). The NLRP3 inflammasome is the most extensively studied one and contains NLRP3, pro-caspase-1, and apoptosis-associated speck-like protein (ASC). The NLRP3 inflammasome can be activated by different stimuli, including damage-associated molecular patterns (DAMPS) and pathogen-associated molecular patterns (PAMPs). The first stimulation is mediated by pro-inflammatory pathways, such as toll like receptor (TLR)-mediated activation of nuclear factor **kB** (NF-**kB**), which upregulates the protein expressions of NLRP3 and pro-IL-1β (Munoz-Planillo et al., 2013; Lu et al., 2014; Abais et al., 2015; Toldo and Abbate, 2018) and reduces the activation threshold of NLRP3 through additional post-translational modifications (Swanson et al., 2019; Yang et al., 2019). The second stimulation includes Ca^2+^ signaling disturbance, K^+^ efflux, ROS production, mitochondrial dysfunction, and lysosomal rupture, which promotes the assembly of inflammasome and activates caspase-1, thus catalyzing the conversion of pro-IL-1β to active IL-1β (Figure 2; Schroder and Tschopp, 2010; He et al., 2016). Activated caspase-1 also cleaves gasdermin D to trigger a specific cell death form named pyroptosis (Shi et al., 2015). Pyroptosis is a new form of pro-inflammatory cell death program and characterized by the pore formation induced by the Gasdermin family and subsequently cellular lysis as well as the release of several pro-inflammatory intracellular cytokines. Two signaling pathways participate in pyroptosis, including caspase-4/5/11 and caspase-1mediated pathways (Shi et al., 2015). The NLRP3 inflammasome is involved in the pathogenesis of many complex diseases, including type 2 diabetes (Hong et al., 2018), atherosclerosis (Grebe et al., 2018), obesity, and gout (Kim et al., 2014). It is reported that the NLRP3 inflammasome also played a vital role in central nervous system (CNS) diseases (Song et al., 2017), including Alzheimer’s disease (AD) (Ising et al., 2019) PD (Saresella et al., 2016; Wang et al., 2019b), and HIV-associated neurocognitive disorders (Walsh et al., 2014). The main neurotoxicity of NLRP3 is the release of IL-1β. The neuroinflammation mediated by IL-1β plays a vital role in CNS diseases, including AD, stroke, multiple dementia, and sclerosis. IL-1β, which is a pleiotropic cytokine, activates microglia and astrocytes to induce the synthesis of other pro-inflammatory and chemotactic mediators in the CNS. Peripherally, IL-1β can induce the expansion of brain-derived T cells (Mendiola and Cardona, 2018).

Autophagy can negatively regulate the activation of the NLRP3 inflammasome by scavenging endogenous activators of inflammasome, including reactive oxygen species (ROS) of damaged mitochondria, inflammatory components, and cytokines. In contrast, NLRP3 inflammasome activation can inhibit autophagy by cleaving signal molecule Toll/IL-1R domain-containing adaptor-inducing IFN-β (TRIF) with caspase-1. The decrease of signal molecule TRIF inhibits autophagy induced by the TLR4-TRIF signaling pathway. The inflammasome activation can also suppress mitophagy in macrophages in a caspase-1-dependent manner, which leads to mitochondrial dysfunction. The interaction between inflammasome and autophagy is necessary to the balance host defense against inflammation and prevent excessive inflammation (Cao et al., 2019; Biasizzo and Kopitar-Jerala, 2020).

It is reported that autophagy plays an important role by influencing the NLRP3 inflammasome in many diseases, including nephropathy, inflammatory bowel disease, inflammatory lung disease, and sepsis (Cao et al., 2019); however, the relevant mechanism has not been fully explained. To study the mechanism of the effects of autophagy on the NLRP3 inflammasome in inflammatory nervous system diseases may provide a new strategy for the treatment of diseases. Therefore, in this review, we are the first to sum up the recent studies about the effects of autophagy on the NLRP3 inflammasome in inflammatory nervous system diseases including AD, Parkinson’s disease, Chronic cerebral hypoperfusion (CCH), depression, cerebral ischemia/reperfusion injury, Early brain injury (EBI) after Subarachnoid hemorrhage (SAH), and Experimental autoimmune encephalomyelitis (EAE) and analyzed the related mechanism to provide reference for future research.

## The Effects of Autophagy on NLRP3 Inflammasome in Alzheimer’s Disease

Alzheimer’s disease is a destructive neurodegenerative disease characterized by extensive loss of neurons and synapsis and gradual loss of memory. The main pathological features of AD are amyloid plaques and neurofibrillary tangles consisting of hyperphosphorylated filaments of the microtubule-associated protein tau. The extracellular accumulation of amyloid beta (β-amyloid) in senile plaques is the main cause of neurodegeneration (Lane et al., 2018). β-amyloid activates the NLRP3 inflammasome to release proinflammatory cytokine IL-1β in microglia. The activated NLRP3 inflammasome leads to neuroinflammation in the brains of AD patients (Bodles and Barger, 2004; Heneka et al., 2013). ASC, a NLRP3 inflammasome component, is elevated in the blood of AD patients and may serve as a biomarker of AD (Scott et al., 2020). Cho et al. (2014) speculate that the autophagy of microglia might be involved in the degradation of extracellular amyloid fibers and play an important role in regulating β-amyloid fiber-mediated inflammatory response. Their research shows that autophagy was induced in microglia by extracellular β-amyloid fibers and involved in the degradation of extracellular β-amyloid fibers. The NLRP3 inflammasome was upregulated in microglia induced by β-amyloid fibers. In addition, the inhibited autophagy enhanced the activation of the NLRP3 inflammasome, indicating that autophagy negatively regulates the NLRP3 inflammasome induced by β-amyloid fibers (Cho et al., 2014). Collectively, it can be deduced that autophagy can eliminate β-amyloid fibers and inhibit the β-amyloid fiber-induced NLRP3 inflammasome to ameliorate AD. Heavy metals, including manganese, are involved in the etiology of neurodegenerative diseases. Manganese (Mn) is an important trace element, which is widely distributed in the earth’s crust. Excessive intake of Mn can lead to neurodegenerative diseases (Iregren, 1999; Erikson and Aschner, 2003). In Mn-induced microglia within the hippocampus of mice, Mn induced NLRP3 inflammasome-mediated inflammation by increasing the protein expression level of the NLRP3 inflammasome and the levels of caspase-1 and IL-1β. Mn treatment also dramatically inhibited autophagy by impairing autophagic lysosomal degradation activity, which mediated the activation of the NLRP3 inflammasome. In conclusion, Mn could induce NLRP3 inflammasome-mediated neuroinflammatory injury in the hippocampus of mice through inhibiting autophagy (Wang et al., 2017). Autophagy-NLRP3 inflammasome is a potential target for the treatment of neurotoxicity caused by heavy metals.

Eicosapentaenoic acid (EPA), mainly in the form of eicosapentaenoic acid-enriched phosphatidylcholine (EPA-PC), is mainly in marine products, including antarctic krill and marine cucumber (Burri et al., 2012). EPA-PC upregulated β-amyloid-suppressed autophagy through promoting the ratio of LC3II/LC3I and autophagosome formation and suppressed β-amyloid-induced the NLRP3 inflammasome to mitigate AD (Wen et al., 2019). Autophagy can clear β-amyloid; therefore, the protective effect of EPA-PC on AD is through inducing autophagy to eliminate β-amyloid-induced NLRP3 inflammasome. Another study shows that the reduction of Beclin 1 and the impairment of autophagy promoted IL-1β and IL-18 release from microglia (Houtman et al., 2019). In addition, microglia isolated from the brains of AD patients showed reduced level of Beclin 1, suggesting that microglial autophagy was impaired in the brains of AD patients (Lucin et al., 2013). Progesterone (PG) is an endogenous neurosteroid, which plays a neuroprotective role in several neurodegenerative disease models (Borowicz et al., 2011; Espinosa-Garcia et al., 2014). It is found that PG can improve the cognitive impairment of AD (Liu et al., 2013; Qin et al., 2015). To elucidate the molecular mechanism of PG neuroprotection, Yang Hong et al. treated astrocytes with PG and found that PG could significantly inhibit β-amyloid-induced neuroinflammatory response and regulated the function of astrocytes by inhibiting ERS and activating autophagy (Hong et al., 2016, 2018). β-amyloid activated NLRP3 inflammasome-mediated inflammation and inhibited autophagy in astrocytes, and PG inhibited β-amyloid-induced activation of NLRP3 inflammasome-mediated inflammation by upregulating astrocyte autophagy (Hong et al., 2019). In conclusion, enhancing autophagy to suppress an NLRP3 inflammasome-mediated neuroinflammatory response is a new strategy for the treatment of AD.

## The Effects of Autophagy on the NLRP3 Inflammasome in Parkinson’s Disease

Parkinson’s disease is the second most common neurodegenerative disease and currently still incurable. It is characterized by major motor dysfunction and other nonmotor symptoms, such as cognitive changes, autonomic nervous dysfunction, sleep disorders. The pathological property of Parkinson’s disease is the progressive loss of dopaminergic neurons and the accumulation of Lewy bodies (LBS) in the neuron with fiber α-synuclein aggregates as the main protein component (Hou et al., 2020; Schwab et al., 2020). New evidence suggests that the low-grade systemic inflammation contributes to the development of degenerative changes in the brain in PD (Wang et al., 2014; Goldberg and Dixit, 2015; Poewe et al., 2017). Therefore, the precise control of excessive neuroinflammation can ameliorate PD. The NLRP3 inflammasome can cause chronic low-degree inflammation (Lu et al., 2014) and is activated in several PD models (Wang et al., 2019b; Haque et al., 2020). Zhou et al. (2016) found that the levels of mature IL-1β and activated caspase-1 in the plasma of PD patients were increased, indicating the inflammation was activated in the pathological process of PD. Consistent with the clinical data, the same results were confirmed in the serum of PD mice. Kaempferol (Ka), a natural polyphenolic small molecule, inhibited neurodegeneration by relieving neuroinflammation in a PD mouse model induced by lipopolysaccharide (LPS). Ka also decreased the protein expression level of the NLRP3 inflammasome and the levels of caspase-1 and IL-1β induced by LPS. The inhibition of Ka disappeared completely in the primary microglia from the NLRP3 knockout PD mouse model, indicating that the NLRP3 inflammasome mediated the neuroprotective effect of Ka. Further mechanism studies show that Ka could counteract LPS-induced inhibition of autophagy and inhibit LPS-induced NLRP3 inflammasome expression by inducing NLRP3 degradation, which were reversed by the autophagy inhibitor (3-MA). Therefore, it can be inferred that Ka inhibits the NLRP3 inflammasome through promoting autophagy. In addition, Ka can promote the ubiquitination of NLRP3. In summary, by inducing NLRP3 ubiquitination-modified degradation, Ka improves LPS-induced NLRP3 inflammasome-related neurodegeneration via promoting NLRP3 degradation through autophagy (Han et al., 2019). Whether Ka can suppress the NLRP3 inflammasome through autophagy via the other ways, such as scavenging reactive oxygen (ROS), needs further study. With the NLRP3 inflammasome and autophagy as targets, Ka has a potential therapeutic effect on PD.

## The Effects of Autophagy on the NLRP3 Inflammasome in Chronic Cerebral Hypoperfusion

Chronic cerebral hypoperfusion, which is a state of chronic cerebral blood flow reduction, is associated with some cerebrovascular and neurodegenerative diseases, such as Alzheimer’s disease (AD) and carotid artery stenosis (Hainsworth and Markus, 2008; Arsava et al., 2018; Choi et al., 2019; Shang et al., 2019). CCH is reported to increase the levels of NLRP3, caspase-1, and IL-1β in the hippocampus and thalamus of AD mice (Shang et al., 2019; Matsuyama et al., 2020). However, the effects of autophagy on the NLRP3 inflammasome in CCH has not been studied. To determine whether autophagy is involved in the activation of the NLRP3 inflammasome and its possible mechanism in CCH, Shao-Hua Su et al. conducted a series of studies and found that CCH caused proinflammatory cytokine release, lysosome dysfunction, and autolysosome accumulation, resulting abnormal autophagy (Su et al., 2017, 2018). Mechanism research revealed that, in rat hippocampus, CCH activated the NLRP3 inflammasome and impaired autophagy, which was significantly attenuated by URB597 (the fatty acid amide hydrolase inhibitor). The autophagy inhibitor 3-MA and lysosome inhibitor CQ could neutralize the effects of URB597 on the CCH-induced NLRP3 inflammasome, suggesting that URB597 alleviated CCH-induced NLRP3 inflammasome activation through promoting restoring CCH-inhibited lysosomal function of autophagy. URB597 could promote CCH-induced defective autophagy by preventing ROS accumulation, and ROS could activate the NLRP3 inflammasome, suggesting that URB597 inhibited CCH-induced NLRP3 inflammasome partly via clearing ROS. In conclusion, URB597 alleviated inflammatory injury by suppressing the CCH-induced NLRP3 inflammasome through promoting autophagy and inhibiting ROS accumulation. Autophagy consisted of three steps, including autophagy formation, transport to lysosome, and degradation in lysosome (Su et al., 2019). Whether the first two participated in CCH-induced NLRP3 inflammasome needs further study.

## The Effects of Autophagy on NLRP3 Inflammasome in Depression

Depression is a chronic recurrent and debilitating mental disease characterized by depression, loss of pleasure, inferiority complex, poor sleep or appetite, and inattention. Depression seriously impairs one’s ability to work or study and even has adverse effects on one’s daily life. In developed countries, depression is the main cause of disability (Smith, 2014; Wang et al., 2020). Increased inflammation is involved in the progression of depression, and the activation of NLRP3 inflammasome in microglia is an important feature of CNS inflammation under chronic stress (Pan et al., 2014; Pariante, 2017). Studies show that the NLRP3 inflammasome level in peripheral blood mononuclear cells is increased in depressive patients (Alcocer-Gomez et al., 2014). In rodents, depression induced by LPS is associated with activation of NLRP3 inflammasome in the brain (Pan et al., 2014). Inflammatory inhibitors have therapeutic effects on depression (Raison et al., 2013). Andrographolide is a diterpenoid lactone with anti-inflammatory and antitumor activities, and it exerts potential neuroprotective effects in diseases of the CNS (Wang et al., 2016; Islam et al., 2018). Andrographolide significantly inhibited inflammatory response in the prefrontal cortex of chronic unpredictable mild stress (CUMS)-induced mice to alleviate depression. In the prefrontal cortex of CUMS mice, Andrographolide inhibited NLRP3 inflammasome-mediated inflammation by decreasing the assembly of the NLRP3 inflammasome and induced autophagy. CQ, an autophagy flux blocker, could attenuate the antidepressant and anti-inflammatory effect of andrographolide, suggesting that andrographolide might ameliorate depression by inhibiting NLRP3 inflammasome-mediated inflammatory injury through inducing autophagy in CUMS mice, which needs further study (Geng et al., 2019). The underlying mechanism of andrographolide in improving depression by affecting autophagy and the NLRP3 inflammasome, including how andrographolide induced autophagy, remains to be elucidated. Autophagy and NLRP3 are potential targets for the treatment of depression.

## The Effects of Autophagy on NLRP3 Inflammasome in Cerebral Ischemia/Reperfusion Injury

Ischemic stroke is still the leading cause of acquired disability and death in adults worldwide. Reperfusion is the main method for the treatment of ischemic stroke, but it can cause serious secondary brain tissue injury, which is called cerebral I/R injury (Donnan et al., 2008; Zhang et al., 2020). Much evidence suggests that inflammation plays a vital role in the occurrence and development of ischemic stroke (Dirnagl et al., 1999). The NLRP3 inflammasome participates in cerebral I/R injury after stroke (Wang et al., 2015; Qiu et al., 2016). Qingkailing alleviates cerebral I/R injury by inhibiting AMPK-mediated activation of NLRP3 inflammation (Ma et al., 2019). Edebenone improves cerebral I/R injury by inhibiting the activity of the NLRP3 inflammasome (Peng et al., 2020). Autophagy and the NLRP3 inflammasome are proven to be related to cerebral I/R injury (Qiu et al., 2016; Sun et al., 2020), but its mechanism is not fully clear. Resveratrol (3,4,5-trihydroxy-trans-stilbene, RSV), a natural polyphenolic compound, is shown to have protective effects in cerebral I/R injury (Burns et al., 2002; Shrikanta et al., 2015; Lu et al., 2020). The results of He et al. (2017) show that resveratrol could improve rat cerebral I/R injury by reducing brain water content and cerebral infarct volume and increasing neurological scores. The mechanism research revealed that RSV suppressed NLRP3 inflammasome-mediated inflammation through decreasing the levels of NLRP3 inflammasome, caspase-1, IL-1β, and IL-18 induced by cerebral I/R injury. Moreover, RSV upregulated Sirt1 expression and promoted autophagy in rat cerebral I/R injury, and 3-MA (an autophagy inhibitor) inhibited autophagy and NLRP3 inflammasome, suggesting that RSV suppressed NLRP3 inflammasome activation through autophagy promotion. Sirt1 siRNA downregulated Sirt1 expression and abolished the effects of RSV on autophagy and NLRP3 inflammasome. Given these results, it can be deduced that RSV ameliorates cerebral I/R injury by inhibiting the NLRP3 inflammasome through autophagy induction via increasing Sirt1expression (He et al., 2017). The Sirt1-AMPK pathway plays a protective role in ischemic stroke (Wang et al., 2011), so whether RSV ameliorates cerebral I/R injury by inhibiting the NLRP3 inflammasome through autophagy induction via Sirt1-AMPK pathway is worth studying. GSK3β is a serine/threonine kinase, which participates in the signal pathway through a phosphorylation-mediated signaling cascade, is activated by phosphorylation. Inactivation of GSK-3β promotes neuronal survival (Zhou et al., 2011; Chen et al., 2016; Chien et al., 2018). The expression of p-GSK-3β was increased in the 24 h following reperfusion after middle cerebral artery occlusion (Chen et al., 2016). GSK-3β siRNA and GSK-3β inhibitor alleviated cerebral I/R injury in rats, demonstrating that inhibition of GSK-3β could alleviate cerebral ischemia/reperfusion injury. The NLRP3 inflammasome played a vital role in cerebral I/R injury, and I/R significantly elevated the levels of the NLRP3 inflammasome, cleaved-caspase-1, IL-1β, and IL-18, which was abrogated by treatment with GSK-3β inhibitor or GSK-3β siRNA, indicating that the inhibition of GSK-3β alleviated I/R-induced brain injury by inhibiting NLRP3 inflammasome-mediated inflammation. The inhibiting GSK-3β could enhance autophagic activity under I/R stimulation, and the autophagy inhibitor could abrogate the effects of the inhibition of GSK-3β on I/R-induced brain injury, which suggested that the suppression of GSK-3β ameliorated cerebral I/R injury in rats by suppressing NLRP3 inflammasome activation via promoting autophagy (Wang et al., 2019c). The signaling pathways of GSK-3β regulating autophagy and NLRP3 are still to be elucidated.

## The Effects of Autophagy on NLRP3 Inflammasome in Early Brain Injury After Subarachnoid Hemorrhage

Subarachnoid hemorrhage is a stroke with high mortality and a high incidence rate (Ciurea et al., 2013). Early brain injury occurs by increasing intracranial pressure and then decreasing cerebral perfusion at the moment of hemorrhage (Schneider et al., 2018). Recent studies show that EBI plays an important role in the poor prognosis of SAH patients. In the past decade, more and more evidence has shown that NLRP3 inflammasome-mediated neuroinflammation promotes the progression of EBI (Sercombe et al., 2002; Dumont et al., 2003; Ostrowski et al., 2006; Chen et al., 2013, 2016; Fann et al., 2013; Cho et al., 2014). Mitophagy is a selective form of autophagy that specifically scavenges damaged mitochondria and can negatively regulate NLRP3 inflammasome (Bienert et al., 2007; Youle and Narendra, 2011). It has been reported that melatonin could improve brain edema by reducing brain water content and attenuated neurological dysfunction by increasing the neurological scores after SAH. Mechanism studies show that melatonin could enhance mitophagy by increasing the LC3-II/LC3-I ratio and the expression levels of mitophagy-associated proteins (PINK1/Parkin) and Atg5, and inhibit NLRP3 inflammasome-mediated inflammation by decreasing the levels of ROS generation, NLRP3 inflammasome, and pro-inflammatory cytokine secretion, and microglial activation induced by SAH. Although 3-mA pretreatment reversed the above effects of melatonin. Moreover, melatonin treatment significantly reduced neuronal cell death induced by SAH. From all above, it could be inferred that melatonin-induced mitophagy protected EBI after SAH by inhibiting the activation of NLRP3 inflammasome (Cao et al., 2017). The relationship between mitophagy and reduced ROS generation needs to be further studied. Whether inflammasome activation affects mitophagy is still to be elucidated.

## The Effects of Autophagy on NLRP3 Inflammasome in Experimental Autoimmune Encephalomyelitis

Multiple sclerosis (MS) is a chronic inflammatory autoimmune disease characterized by immune-mediated demyelination and neurodegeneration of the CNS. EAE is an animal model of MS that has been widely studied in recent years (Zepp et al., 2011). Cannabinoid receptor 1 (CB2R) is mainly expressed in immune cells intimately and has been reported to be related to the inflammation in MS. A large number of studies show that CB2R and the NLRP3 inflammasome play an important role in the development of EAE (Rossi et al., 2011; Lou et al., 2012; Inoue and Shinohara, 2013). Activating CB2R could ameliorate clinical symptoms and leukocyte infiltration in EAE. CB2R-deficiency notably increased NLRP3 expression and the secretion of IL-1β and activated Casp-1 activation in EAE, and HU-308 (CB2R agonist) had the opposite effects, indicating that CB2R inhibited NLRP3 inflammasome-mediated inflammation. CB2R-deficiency also decreased the levels of LC3-II/LC3-I ratio and Beclin 1 in EAE, and HU-308 had the opposite effects, suggesting that CB2R promoted autophagy. Inhibition of autophagy with ATG5 siRNA attenuated the inhibitory effect of HU-308 on the NLRP3 inflammasome, suggesting that the induction of autophagy mediates, at least partly, the inhibitory effect of CB2R on NLRP3 inflammasome formation. Collectively, activation of CB2R can improve EAE through suppression of NLRP3 inflammasome via upregulating autophagy, which provides a good strategy to treat MS (Shao et al., 2014). The mechanism of autophagy inhibiting NLRP3 inflammasome in EAE remains to be studied. Contrary to the above conclusion, there has been a report that inhibition of autophagy of dendritic cells attenuated inflammatory infiltration in EAE mice (Bhattacharya et al., 2014), which might be due to the different species of target cells.

## Conclusion

**FIGURE 3:**
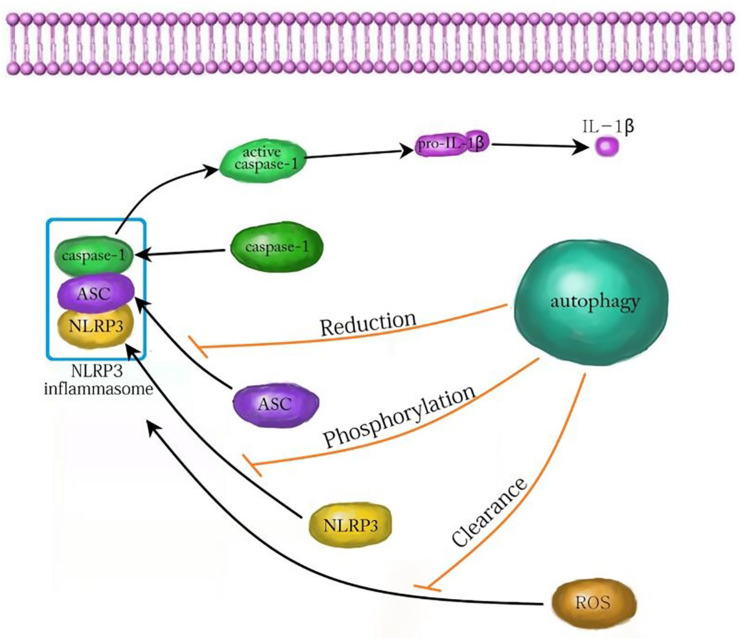
The mechanism of autophagy inhibiting NLRP3 inflammasome. Autophagy can inhibit the activation of the NLRP3 inflammasome by decreasing ASC, increasing NLRP3 phosphorylation and clearing ROS. ASC, apoptosis associated speck like protein; ROS, reactive oxygen species.

In conclusion, autophagy plays an important role in the development and treatment of many nervous system diseases by affecting NLRP3 inflammasome. The relevant mechanism is very complex and needs to be further clarified. At present, most studies confirm that autophagy attenuates inflammatory injury by inhibiting the NLRP3 inflammasome. Autophagy inhibits the NLRP3 inflammasome by reducing ASC, phosphorylating NLRP3, and scavenging ROS (Figure 3; Cao et al., 2019). In contrast, autophagy also promotes the NLRP3 inflammasome in yeast cells (Dupont et al., 2011). Whether autophagy can promote the NLRP3 inflammasome in mammalian cells needs to be further explored. Although autophagy plays an important role by regulating the NLRP3 inflammasome in neuroinflammatory injury, there are still many problems to be solved. For example, can autophagy affect NLRP3 inflammasome through other pathways? Are there side effects of promoting autophagy to inhibit inflammatory injury? With the continuous progress of research, targeting autophagy and NLRP3 inflammation may offer a new way for the treatment of inflammatory nervous system diseases.

## Author Contributions

HW: devising, writing, and funding this manuscript. SZ: writing and funding this manuscript. XL: drawing. JW: writing. All authors contributed to the article and approved the submitted version.

## Conflict of Interest

The authors declare that the research was conducted in the absence of any commercial or financial relationships that could be construed as a potential conflict of interest.
